# Two‐year follow‐up of a randomized phase III clinical trial of nivolumab vs. the investigator's choice of therapy in the Asian population for recurrent or metastatic squamous cell carcinoma of the head and neck (CheckMate 141)

**DOI:** 10.1002/hed.26331

**Published:** 2020-06-24

**Authors:** Chia‐Jui Yen, Naomi Kiyota, Nobuhiro Hanai, Shunji Takahashi, Tomoya Yokota, Shigemichi Iwae, Yasushi Shimizu, Ruey‐Long Hong, Masahiro Goto, Jin‐Hyoung Kang, Wing Sum Kenneth Li, Robert L. Ferris, Maura Gillison, Toshimitsu Endo, Vijayvel Jayaprakash, Makoto Tahara

**Affiliations:** ^1^ Division of Hematology and Oncology, Department of Internal Medicine National Cheng Kung University Hospital, College of Medicine, National Cheng Kung University Tainan Taiwan; ^2^ Department of Medical Oncology and Hematology Kobe University Hospital Kobe Japan; ^3^ Cancer Center Kobe University Hospital Kobe Japan; ^4^ Department of Head and Neck Surgery Aichi Cancer Center Hospital Nagoya Japan; ^5^ Department of Medical Oncology Cancer Institute Hospital, Japanese Foundation for Cancer Research Tokyo Japan; ^6^ Division of Gastrointestinal Oncology Shizuoka Cancer Center Shizuoka Japan; ^7^ Department of Head and Neck Surgery Hyogo Cancer Center Akashi Japan; ^8^ Department of Medical Oncology Hokkaido University Hospital Sapporo Japan; ^9^ Department of Oncology National Taiwan University Hospital Taipei Taiwan; ^10^ Cancer Chemotherapy Center Osaka Medical College Hospital Takatsuki Japan; ^11^ Division of Medical Oncology, Department of Internal Medicine The Catholic University of Korea, Seoul St. Mary's Hospital Seoul Republic of Korea; ^12^ Department of Clinical Oncology Queen Elizabeth Hospital Hong Kong China; ^13^ UPMC Hillman Cancer Center University of Pittsburgh Medical Center Cancer Center Pittsburgh Pennsylvania USA; ^14^ Department of Thoracic Head and Neck Medical Oncology, Division of Cancer Medicine MD Anderson Cancer Center Houston Texas USA; ^15^ Section 1, Oncology Medical Affairs ONO Pharmaceutical Co., Ltd Osaka Japan; ^16^ Oncology Clinical Development BMS, Bristol‐Myers Squibb Princeton New Jersey USA; ^17^ Department of Head and Neck Medical Oncology National Cancer Center Hospital East Kashiwa Japan

**Keywords:** Asian population, clinical trial, immunotherapy, nivolumab, squamous cell carcinoma of the head and neck

## Abstract

**Background:**

The present study evaluated the 2‐year survival of the Asian population in the CheckMate 141 trial.

**Methods:**

The CheckMate 141 trial included patients with recurrent or metastatic (R/M) squamous cell carcinoma of the head and neck (SCCHN). In the present study, 34 Asian patients (nivolumab group: 23 patients; investigator's choice of therapy [IC] group: 11 patients) were analyzed.

**Results:**

The median overall survival (OS) was 12.1 and 6.2 months for the nivolumab and IC groups, respectively. The estimated 2‐year OS rates were 22.7% and 0% for the nivolumab and IC groups, respectively. In the nivolumab group, the patients with any treatment‐related adverse events (TRAEs), including skin‐related disorders, showed better OS than the patients without any TRAEs.

**Conclusions:**

Nivolumab demonstrated prolonged OS benefits in the Asian population with platinum‐refractory R/M SCCHN and a favorable safety profile. TRAEs, including skin‐related disorders, may be favorable prognostic factors for nivolumab efficacy.

**Clinical trial registration:**

NCT02105636.

**Abbreviations**
N/A: not applicable
PR: partial response
SD: stable disease
PD: progressive disease

## 1 INTRODUCTION

Squamous cell carcinoma of the head and neck (SCCHN) is one of the most common cancers worldwide, such that >800 000 new cases of SCCHN and >400 000 deaths caused by SCCHN were reported in 2018.^1^ In Asia, including Japan, Taiwan, Korea, and Hong Kong, consistent increases in the number of patients diagnosed with SCCHN have been reported.^2^, ^3^, ^4^, ^5^

Platinum‐based chemotherapy with cetuximab is one of the first‐line standard treatment options for recurrent or metastatic(R/M) SCCHN; however, patients receiving this treatment have poor prognosis, and limited treatment options exist in the second‐line setting.^6^ The median overall survival (OS) rate for SCCHN is <6 months, particularly for patients who have experienced recurrence within 6 months of treatment with first‐line platinum‐based chemotherapy.^7^, ^8^ Recently, pembrolizumab alone or in combination with chemotherapy has been added as new first‐line treatment options for patients with R/M SCCHN post‐platinum therapy.^9^ The CheckMate 141 trial is a global Phase III clinical trial of nivolumab vs the investigator's choice of therapy (IC) for platinum‐refractory R/M SCCHN. Based on the positive results of the CheckMate141 trial, nivolumab was approved in the United States in 2016 for patients with R/M SCCHN. Subsequently, it was approved in Japan, Taiwan, and Korea in 2017, but it has not been approved in Hong Kong at the time of publication.

As observed with a 2‐year follow‐up of the global population in the CheckMate 141 trial, nivolumab demonstrated prolonged OS benefits over IC, with a manageable safety profile.^10^, ^11^ In addition, some post‐hoc analyses of the CheckMate 141 trial on tumor programmed death ligand 1 (PD‐L1) status,^11^ human papillomavirus (HPV),^11^ health‐related quality of life (QOL),^12^ first‐line therapy,^13^ age,^14^ and prior use of cetuximab^15^ revealed that nivolumab resulted in prolonged OS benefits compared with IC in all sub‐populations. Therefore, nivolumab has become the standard second‐line treatment for R/M SCCHN following the first‐line platinum‐based chemotherapy.

We have previously reported the results of a 2.3‐month minimum follow‐up analysis of parameters, including efficacy, safety, and QOL, in the Asian population in the CheckMate 141 trial.^16^ The median OS was numerically higher in the Asian population than in the global population, whereas the median progression‐free survival (PFS) rate was similar. The most commonly reported treatment‐related adverse events (TRAEs) in the Asian as well as global population were skin‐related disorders such as pruritus, rash, and maculopapular rash. The present analysis aimed to provide a 2‐year OS update of the CheckMate 141 trial involving the Asian population based on a 24.2‐month minimum follow‐up. Further, we report the relationship between the efficacy and safety of nivolumab in the Asian population.

## 2 MATERIALS AND METHODS

### 2.1 Study design and patients

This Phase III, open‐label, randomized trial included patients from 55 sites in 15 countries (including 8 sites in Japan, 2 in Taiwan, 1 in Korea, and 1 in Hong Kong). An interactive voice response system was used to randomize patients. After stratification by prior cetuximab treatment (yes/no), the patients were randomized at a ratio of 2:1 to receive either nivolumab (nivolumab group) or the IC (methotrexate, docetaxel, or cetuximab; IC group). The treatments administered were as follows: nivolumab monotherapy, which was administered at a dose of 3 mg/kg at 2‐week intervals; cetuximab at a dose of 400 mg/m^2^ IV once, which was then decreased to a dose of 250 mg/m^2^ at 1‐week intervals; methotrexate at a dose of 40 mg/m^2^ IV push at 1‐week intervals, which was then increased to a dose of 60 mg/m^2^ (if tolerated) and as per local practices; and docetaxel at a dose of 30 mg/m^2^ IV at 1‐week intervals, which was then increased to a dose of 40 mg/m^2^ if tolerated and as per local practices. Patient crossover was prohibited until the intermediate analysis was completed.^10^ Treatment was continued until intolerable toxicity or disease progression was observed (nivolumab could be continued beyond initial disease progression based on the investigator‐assessed benefits).

The CheckMate 141 trial was carried out according to Good Clinical Practice guidelines and approved by the Institutional Review Board or Independent Ethics Committee at each center prior to study commencement. The study was conducted with the ethical principles in the Declaration of Helsinki (version 2002). All patients provided written informed consent prior to entering the study. Full study methodology of the CheckMate 141 trial has been described previously.^10^

### 2.2 Outcomes

The primary endpoint of the study was OS, which was defined as the time from randomization to the date of death due to any cause. The patients were followed up at 3‐month intervals until death, withdrawal of consent, or loss to follow‐up. The secondary endpoints of the study included PFS (time from randomization to the date of disease progression or death) and objective response rate (ORR), which was evaluated using RECIST v1.1. In addition, acute toxic effects were evaluated according to the Common Terminology Criteria for Adverse Events (version 4.0) at each treatment visit until 100 days after the last treatment dose. Select adverse events (AEs) were defined as AEs with potential immunological causes. Although dose modifications were not permitted for nivolumab, they were allowed for methotrexate, cetuximab, and docetaxel on the basis of the type and grade of toxicity.

### 2.3 Statistical analyses

The analyses in this study are based on a September 4, 2017, data cut‐off and represent a minimum follow‐up of 24.2 months. Treatment efficacy was analyzed in the intent‐to‐treat population, whereas treatment safety was analyzed in patients who received a minimum of one treatment dose. OS, PFS, and post‐progression survival (PPS) were evaluated using the Kaplan‐Meier method. Hazard ratios (HRs) and the corresponding 2‐sided 95% CIs were determined using the Cox proportional hazards model. In this analysis, PPS was defined as time from the progressive disease (PD) of first response to death. The SAS version 9.4 (SAS Institute Inc., Cary, North Carolina) was used for all statistical analyses.

## 3 RESULTS

### 3.1 Patients and treatment

The baseline characteristics of the 34 Asian patients have been described previously.^16^ Briefly, the population in the nivolumab group was mostly male (91.3%) with an Eastern Cooperative Oncology Group (ECOG) PS of 1 (82.6%). The population in the IC group was also mostly male (90.9%) with an ECOG PS of 1 (90.9%). The patients aged ≥75 years old were not included in both groups in the Asian population. The Asian population included patients from Japan (n = 27), Hong Kong (n = 1), Taiwan (n = 5), and Korea (n = 1). The patients were randomized to receive nivolumab (n = 23) or IC (n = 11). The patients in the IC group received methotrexate (n = 8), docetaxel (n = 2), or cetuximab (n = 1). At the time of analysis, no patient remained on nivolumab treatment or IC. The swimmer plots of the 34 Asian patients are shown in Figure S1 and include the time periods of subsequent chemotherapy after nivolumab treatment or IC. Among the patients who received subsequent chemotherapy, 15 (65.2%) belonged to the nivolumab group and 5 (45.5%) to the IC group.

### 3.2 Efficacy

#### 3.2.1 Overall survival (OS)

**FIGURE 1:**
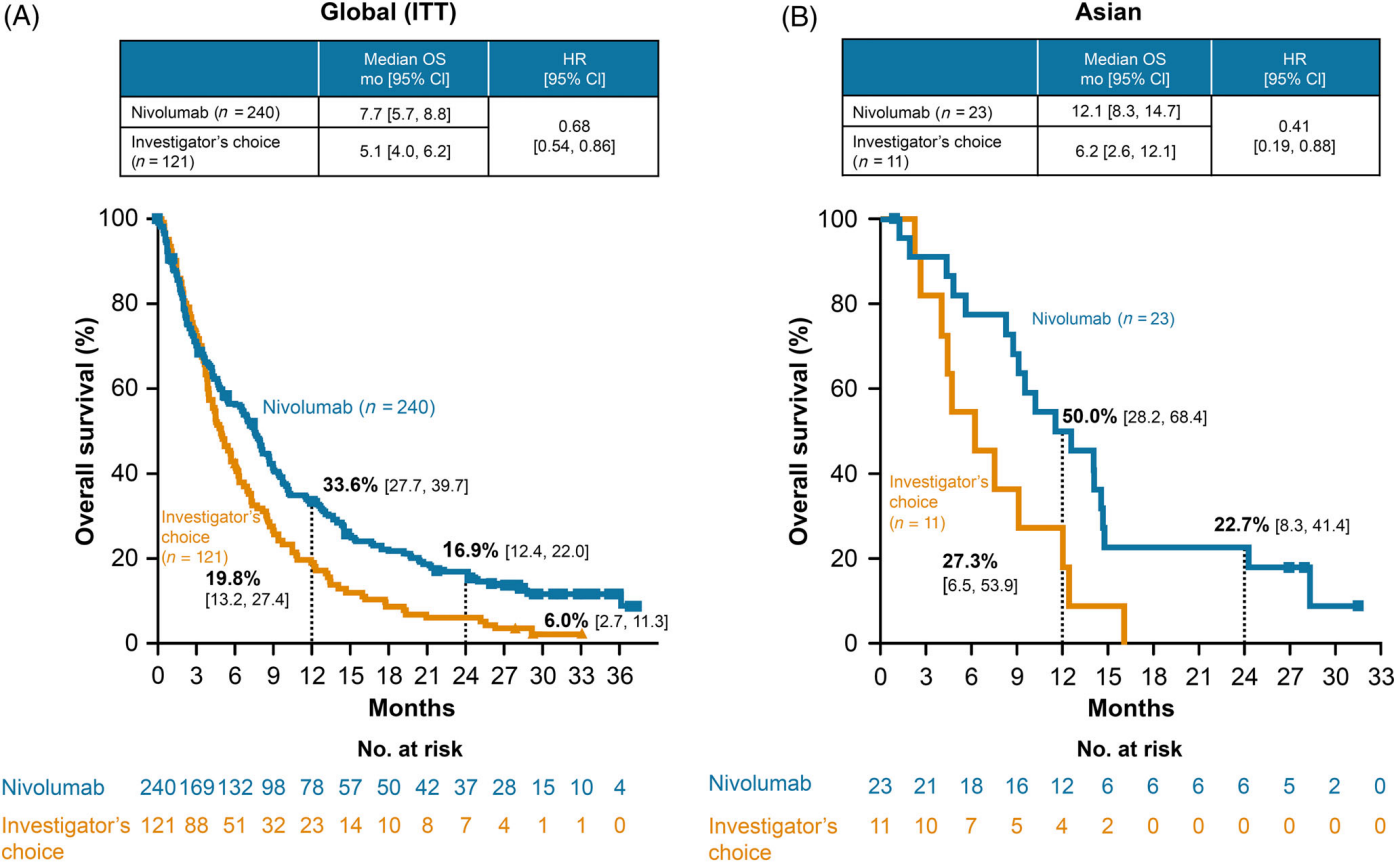
Overall survival (OS) in the global population, A, and Asian population, B. A, is reproduced with permission from Oral Oncology.^11^ Abbreviations: CI, confidence interval; HR, hazard ratio; ITT, intention to treat [Color figure can be viewed at wileyonlinelibrary.com]

At the minimum follow‐up of 24.2 months, among the 34 Asian patients, 3 (13%) in the nivolumab group and 0 (0%) in the IC group had survived. The OS rates in the global and Asian populations are shown in Figure 1A,B. The median OS of the Asian patients was 12.1 months (95% CI 8.3‐14.7) in the nivolumab group and 6.2 months (95% CI 2.6‐12.1) in the IC group. The HR for risk of death with nivolumab treatment vs IC was 0.41 (95% CI 0.19‐0.88). The estimated 1‐year OS rate with nivolumab treatment was 50.0% (95% CI 28.2‐68.4), whereas that with IC was 27.3% (95% CI 6.5‐53.9). The estimated 2‐year OS rates were 22.7% (95% CI 8.3‐41.4) and 0% in the nivolumab and IC groups, respectively.

#### 3.2.2 Progression‐free survival (PFS)

The median PFS in the Asian population was similar in both the groups: 1.9 months (95% CI 1.6‐7.5) in the nivolumab group and 1.8 months (95% CI 0.4‐6.1) in the IC group. The HR was 0.56 (95% CI 0.24‐1.29) (Figure S2). Both the estimated 1‐year and 2‐year PFS rates were 12.7% (95% CI 2.5‐31.4) in the nivolumab group. The estimated 1‐year PFS rate was 0% in the IC group.

#### 3.2.3 Best overall responses (BORs)

The BOR, time to response, and duration of response in the global and Asian populations are summarized in Table 1. Chronological changes in tumor diameters were monitored using RECIST v1.1 (Figure S3). In the Asian population, the BORs remained unchanged from that reported by the previous study,^16^ with 6 partial responses (PRs) (26.1%) observed in the nivolumab group and no objective response observed in the IC group. The baseline characteristics of the six patients with PR, including time after initiation of nivolumab treatment, time of receiving nivolumab treatment, follow‐up time after nivolumab treatment, and regimen of the subsequent chemotherapy, are summarized in Table S1. There were no obvious correlations among these parameters, but it should be noted that five of the six patients with PR survived for >2 years after initiation of nivolumab treatment, including the period of subsequent chemotherapy.

**TABLE 1:** Best overall response in the global and Asian populations

	Global^11^ ^,^ ^a^		Asian	
	Nivolumab	IC	Nivolumab	IC
	(n = 240)	(n = 121)	(n = 23)	(n = 11)
Best overall response, n (%)				
Complete response	7 (2.9)	1 (0.8)	0	0
Partial response	25 (10.4)	6 (5.0)	6 (26.1)	0
Stable disease	55 (22.9)	43 (35.5)	1 (4.3)	4 (36.4)
Progressive disease	99 (41.3)	42 (34.7)	16 (69.6)	6 (54.5)
Unable to determine	54 (22.5)	29 (24.0)	0	0
ORR, n (%)	32 (13.3)	7 (5.8)	6 (26.1)	0
(95% CI)	[9.3‐18.3]	[2.4‐11.6]	[10.2‐48.4]	[0‐28.5]
Time to objective response among responders, median (range) (months)	2.1 (1.8‐7.4)	2.0 (1.9‐4.6)	2.6 (1.9‐6.0)	N/A
Duration of response among responders, median (range) (months)	9.7 (2.8 to 32.8+)	4.0 (1.5+ to 11.3)	7.8 (5.6 to 10.2)	N/A

Abbreviations: CI, confidence interval; IC, investigator's choice of therapy; NR, not reached; ORR, objective response rate; N/A, not applicable.

a The global population includes Asian patients. These results are reproduced with permission from Oral Oncology.^11^

#### 3.2.4 Post progression survival (PPS)

In the Asian population, 16 of the 23 patients (69.6%) in the nivolumab group and 6 of the 11 patients (54.5%) in the IC group displayed PD (Table 1). Among them, 11 of the 16 patients (68.8%) in the nivolumab group and 3 of the 6 patients (50.0%) in the IC group received subsequent chemotherapy. The PPS rates are presented in Figure S4. The median PPS time was 7.9 months (95% CI 3.0‐10.9) in the nivolumab group and 4.5 months (95% CI 1.8‐14.4) in the IC group. The HR for risk of death after progression with nivolumab treatment vs IC was 0.87 (95% CI 0.31‐2.47). The estimated 6‐month PPS rate was 67.3% (95% CI 38.3‐84.9) in the nivolumab group and 16.7% (95% CI 0.8‐51.7) in the IC group.

### 3.3 Safety

#### 3.3.1 Treatment‐related adverse events (TRAEs)

In the Asian population, the TRAEs that occurred with a frequency of >10% for any group are shown in Table 2. TRAEs were reported in 17 patients (73.9%) in the nivolumab group and in 10 patients (90.9%) in the IC group. The major TRAEs reported in the nivolumab group were decreased appetite, pruritus, rash, fatigue, and hypothyroidism, whereas those reported in the IC group were nausea, stomatitis, and decreased appetite. Skin‐related TRAEs were the most common select TRAEs in the nivolumab group (47.8%, 11 patients): pruritus (5 patients), rash (4 patients), and maculopapular rash (2 patients). No newly observed grade 3‐4 TRAEs were reported from the 2‐year long‐term survival update in the Asian population.

**TABLE 2:** Incidence of treatment‐related adverse events (TRAEs) occurring in 10% of patients in any group, and of select TRAEs by category in the Asian populations

	Nivolumab (n = 23)		IC (n = 11)	
	Any grade	Grade 3–4	Any grade	Grade 3‐4
Any TRAE, n (%)	17 (73.9)	2 (8.7)	10 (90.9)	3 (27.3)
Fatigue	6 (26.1)	0	0	0
Decreased appetite	5 (21.7)	0	3 (27.3)	0
Pruritus	5 (21.7)	0	0	0
Rash	4 (17.4)	0	0	0
Hypothyroidism	3 (13.0)	0	0	0
Nausea	2 (8.7)	0	3 (27.3)	0
Stomatitis	1 (4.3)	0	3 (27.3)	0
Diarrhea	1 (4.3)	0	2 (18.2)	0
Anemia	0	0	2 (18.2)	2 (18.2)
Epistaxis	0	0	2 (18.2)	0
Select TRAE, n (%)				
Skin	11 (47.8)	0	1 (9.1)	0
Endocrine	4 (17.4)	0	0	0
Gastrointestinal	1 (4.3)	0	2 (18.2)	0
Hypersensitivity/infusion reaction	0	0	1 (9.1)	0

*Note:* Includes events reported between the first dose and 30 days after the last dose of the study treatment.

Abbreviation: IC, investigator's choice of therapy.

#### 3.3.2 Relationship between efficacy and safety of nivolumab

**FIGURE 2:**
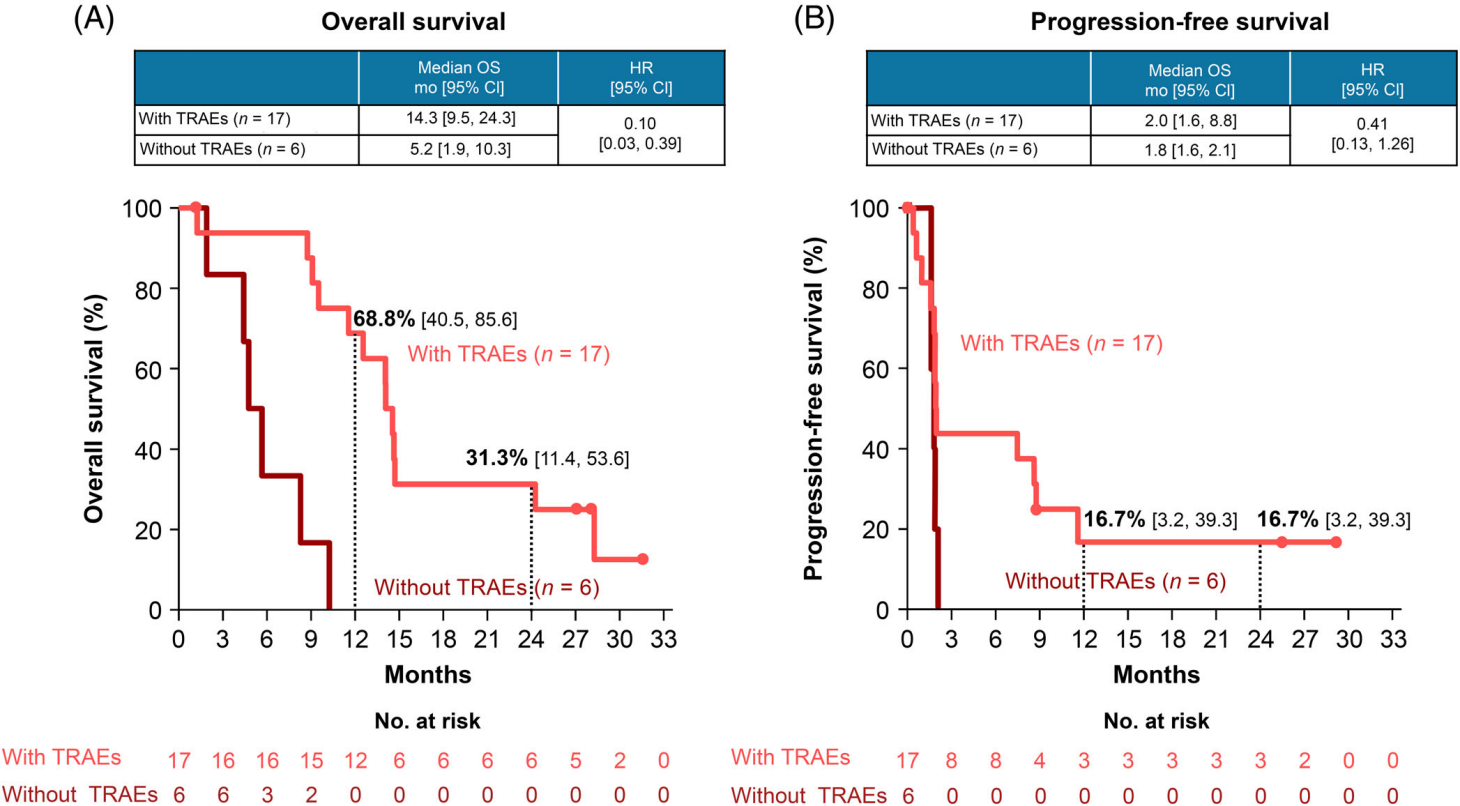
Overall survival (OS), A, and progression‐free survival (PFS), B, in patients with and without treatment‐related adverse events (TRAEs). Abbreviations: CI, confidence interval; HR, hazard ratio [Color figure can be viewed at wileyonlinelibrary.com]

TRAEs stratified by responders (PR and CR) and nonresponders (SD and PD) are shown in Table 3. All the six responders to nivolumab treatment presented with any grade TRAEs, with the skin (100%) and endocrine system (16.7%) being the most affected sites. Conversely, 11 of the 17 (64.7%) nonresponders to nivolumab treatment presented with any grade TRAEs, with the skin (29.4%), endocrine system (17.6%), and gastrointestinal system (5.9%) being the most affected sites. In the IC group, all the 10 patients were considered nonresponders and had any grade TRAEs. The OS and PFS of the patients in the nivolumab group with or without TRAEs are shown in Figure 2A,B. The median OS was 14.3 months (95% CI 9.5‐24.3) for the patients with TRAEs and 5.2 months (95% CI 1.9‐10.3) for the patients without TRAEs (HR 0.10, 95% CI 0.03‐0.39). The estimated 2‐year OS rates were 31.3% (95% CI 11.4‐53.6) and 0% for the patients with and without TRAEs, respectively. The median PFS was 2.0 months (95% CI 1.6‐8.8) and 1.8 months (95% CI 1.6‐2.1) for the patients with and without TRAEs, respectively (HR 0.41, 95% CI 0.13‐1.26).

**TABLE 3:** Treatment‐related adverse events (TRAEs) by responders and nonresponders

	Responders (CR/PR)				Nonresponders (SD/PD)			
	Nivolumab		IC		Nivolumab		IC	
	(n = 6)		(n = 0)		(n = 17)		(n = 10)	
	Any grade	Grade 3‐4	Any grade	Grade 3‐4	Any grade	Grade 3‐4	Any grade	Grade 3‐4
Any TRAE, n (%)	6 (100)	1 (16.7)	0	0	11 (64.7)	1 (5.9)	10 (100)	3 (30)
Select TRAE, n (%)								
Skin	6 (100)	0	0	0	5 (29.4)	0	1 (10.0)	0
Endocrine	1 (16.7)	0	0	0	3 (17.6)	0	0	0
Gastrointestinal	0	0	0	0	1 (5.9)	0	2 (20.0)	0
Hypersensitivity/infusion reaction	0	0	0	0	0	0	1 (10.0)	0

Abbreviations: IC, investigator's choice of therapy; CR, complete response; PR, partial response; SD, stable disease; PD, progressive disease.

**FIGURE 3:**
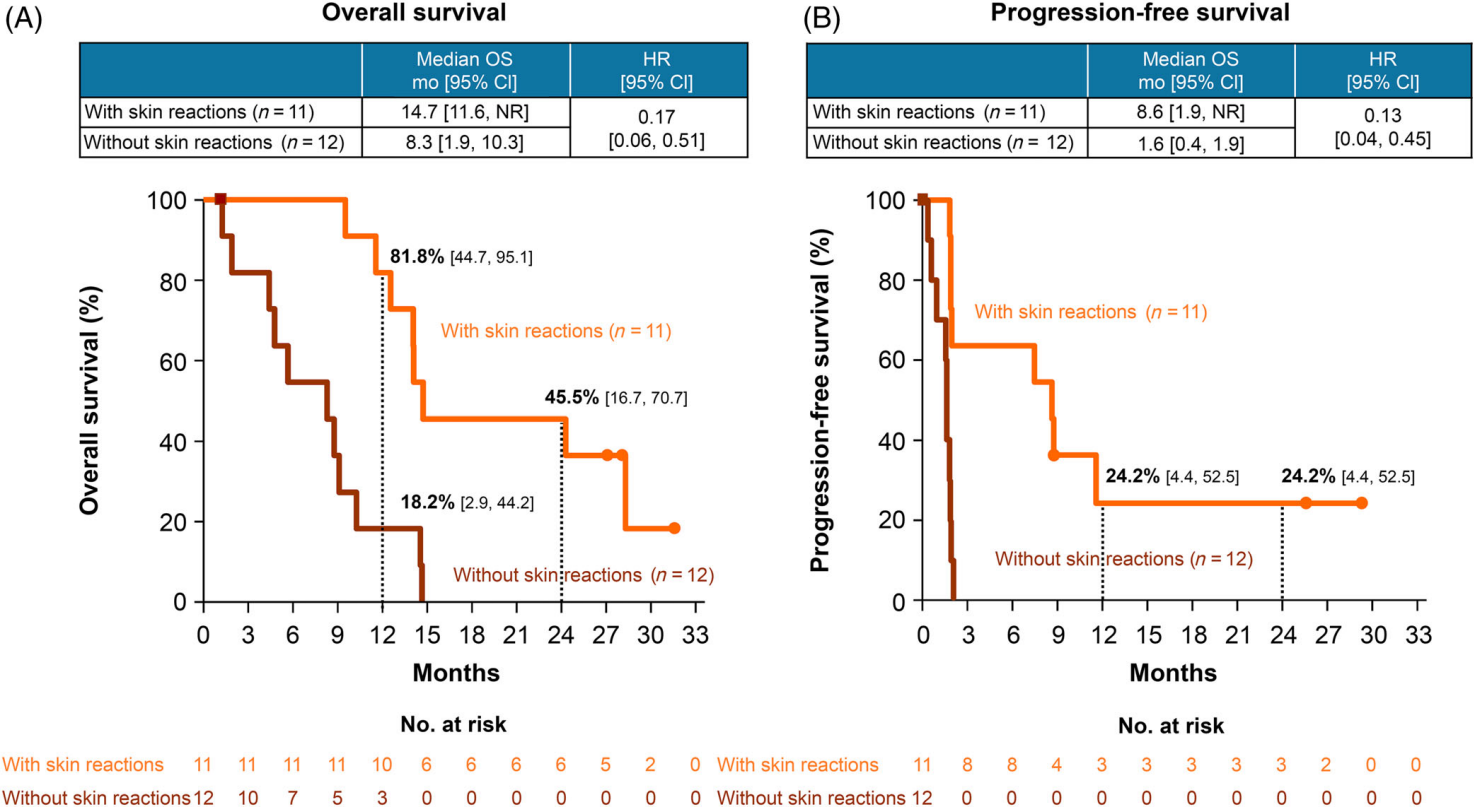
Overall survival (OS), A, and progression‐free survival, B, in patients with/without skin reactions in the Asian population. Abbreviations: CI, confidence interval; HR, hazard ratio; NR, not reached [Color figure can be viewed at wileyonlinelibrary.com]

Finally, the OS and PFS of patients with or without skin‐related TRAEs are shown in Figure 3A,B. The median OS was 14.7 months (95% CI 11.6‐NR) and 8.3 months (95% CI 1.9‐10.3) for the patients with and without skin‐TRAEs, respectively (HR 0.17, 95% CI 0.06‐0.51). The estimated 2‐year OS rates were 45.5% (95% CI 16.7‐70.7) and 0% and the median PFS was 8.6 months (95% CI 1.9‐NR) and 1.6 months (95% CI 0.4‐1.9) for the patients with and without skin‐related TRAEs, respectively (HR 0.13, 95% CI 0.04‐0.45).

## 4 DISCUSSION

In this study, a 2‐year follow‐up of the CheckMate141 trial was analyzed to demonstrate the potential OS benefits of nivolumab treatment compared with IC in Asian patients with platinum‐refractory R/M SCCHN. The HR for risk of death with nivolumab vs IC trended lower with longer follow‐up (1‐year follow‐up: 0.50 [95% CI 0.17‐1.48]; 2‐year follow‐up: 0.41 [95% CI 0.19‐0.88]). These results are consistent with those reported for the global population (1‐year follow‐up: 0.70 [97.73% CI 0.51‐0.96]; 2‐year follow‐up: 0.68 [95% CI 0.54‐0.86])^10^, ^11^ and suggest the long‐term clinical benefits of nivolumab in both the populations. According to the safety profile, two patients reported fatigue, one reported rash, and two reported newly identified hypothyroidism at the 2‐year follow‐up compared with the 1‐year follow‐up results in the Asian population. However, the TRAE grade was ≤2 in all these five patients, with no newly identified grade 3 to 4 TRAEs from the previous data cut‐off in the Asian patients. Thus, similar to that in the global population, nivolumab has maintained a favorable safety profile in the Asian population. In addition, recent studies have reported the long‐term clinical benefits and favorable safety profile of nivolumab monotherapy in the Asian and Japanese populations as well as in the global population for various types of cancers, including melanoma,^17^, ^18^ non‐small cell lung carcinoma (NSCLC),^19^, ^20^ and renal cell carcinoma (RCC).^21^, ^22^ Taken together, these results suggest that nivolumab has long‐term clinical benefits for both Asian and global populations against various cancers such as platinum‐refractory R/M SCCHN.

In the past several years, studies have reported that subsequent chemotherapy after immunotherapy may be effective in patients with NSCLC.^23^, ^24^ Indeed, positive results have been reported for this strategy in patients with R/M SCCHN.^25^, ^26^ In the present study, we evaluated whether the OS benefits observed in the Asian patients treated with nivolumab were associated with subsequent chemotherapy. From the analysis of the responders with nivolumab (Table S1), five of six patients survived >2 years. Among them, three patients received subsequent chemotherapy for >1 year. Therefore, this responder analysis also added positive information for the effectiveness of subsequent chemotherapy. Furthermore, PPS analysis revealed a possible advantage of prolonged survival in the patients in the nivolumab group who received subsequent chemotherapy (Figure S4). Thus, although the sample size of this study limits any definitive conclusions, nivolumab may contribute to potentiate the benefit of subsequent chemotherapy.

Regarding the relationship between the efficacy and safety of nivolumab, studies on patients with melanoma,^27^ NSCLC,^20^, ^28^, ^29^ RCC,^30^ and gastric cancer^31^ have reported that the occurrence of immune‐related AEs was significantly associated with clinical outcomes in patients treated with nivolumab. Similarly, in patients with R/M SCCHN, recent studies have reported that the occurrence of TRAEs was associated with a better prognosis.^32^, ^33^ In the present study, all of the responders (CR and PR) presented with any grade TRAE, whereas the incidence rate of any grade TRAE in the nonresponders was only 64.7% (Table 3). Furthermore, the median OS and 2‐year OS rates were higher in patients with TRAEs than in patients without TRAEs (Figure 2A). Therefore, consistent with the findings of previous reports,^32^, ^33^ a correlation between TRAEs and a better prognosis is expected. Because the most frequent category of TRAEs that occurred with nivolumab treatment in the Asian population was skin‐related disorders (47.8%), the OS and PFS in the patients with and without skin‐related disorders were examined. As a result, there were remarkable differences in the OS and PFS between patients who presented with and without skin‐related disorders (Figure 3A,B). This has also been observed in patients with melanoma^27^, ^34^ and NSCLC.^28^, ^29^ Therefore, it is possible that skin‐related disorders caused by nivolumab treatment serve as a viable predictor of better prognosis. To explore this possibility, we examined the relationship between the onset of skin‐related disorders and that of the first response to nivolumab treatment. However, swimmer plots showed various timing of onsets of skin‐related disorders (Figure S5), suggesting that skin‐related disorders are prognostic for the efficacy of nivolumab.

It should be noted that approximately 50% of the Asian patients treated with nivolumab had skin‐related disorders (Table 3), whereas only 17.4% of the global patients had any incidence of skin‐related disorders.^11^ A possible reason for this difference is the small sample size (23 patients) in this analysis. However, although further studies with larger sample sizes are warranted to make appropriate conclusions, the current results suggest that Asian patients develop skin‐related TRAEs during and after nivolumab treatment at a higher rate. Consistent with this notion, studies on the 3‐year follow‐up of the CheckMate 017/057 and ONO‐7643‐05/06 trials reported that the incidence rate of skin‐related disorders, including rash and pruritus, was higher in the Japanese population than in the global population.^19^, ^20^ Moreover, according to the analysis from KEYNOTE‐012, which is a clinical trial for patients with R/M SCCHN, the incidence of rash was higher in Asian patients receiving pembrolizumab than in the global patients.^35^, ^36^ Thus, regardless of whether the patient is considered a responder or nonresponder, all patients, particularly Asian patients, should be carefully monitored for the development of skin reactions during and after nivolumab treatment.

The present study had some limitations. One major limitation of this study was the small number of Asian patients, which made detailed comparisons between the treatments difficult. Accordingly, future trials should be conducted in larger populations of Asian patients to adequately determine the efficacy and safety of nivolumab in this patient group.

In conclusion, based on the 2‐year follow‐up data of the CheckMate 141 trial, nivolumab demonstrated a prolonged OS benefit over IC and a favorable long‐term safety profile in Asian patients with platinum‐refractory R/M SCCHN. TRAEs, including skin‐related disorders, may be favorable prognostic factors for the efficacy of nivolumab.

## CONFLICT OF INTEREST

Dr Yen reports grants from ONO Pharmaceutical and Bristol‐Myers Squibb. Dr Kiyota reports grants from ONO Pharmaceutical, AstraZeneca, Pfizer, and Chugai Pharmaceutical; honoraria from ONO Pharmaceutical, Bristol‐Myers Squibb, Merck Serono, Eisai, and Bayer. Dr Hanai reports grants from ONO Pharmaceutical and Bristol‐Myers Squibb. Dr Takahashi reports grants from ONO Pharmaceutical and Bristol‐Myers Squibb. Dr Yokota reports grants from ONO Pharmaceutical, Bristol‐Myers Squibb, AstraZeneca, Merck Biopharma, MSD, Novartis, Incyte, Eli Lilly, Eisai, and Chugai Pharmaceutical. Dr Iwae reports grants from ONO Pharmaceutical and Bristol‐Myers Squibb. Dr Shimizu reports grants from ONO Pharmaceutical and Bristol‐Myers Squibb. Dr Hong reports grants from ONO Pharmaceutical and Bristol‐Myers Squibb. Dr Goto reports grants from Bristol‐Myers Squibb; and grants, personal fees, and nonfinancial support from ONO Pharmaceutical; and grants, personal fees, and nonfinancial support from Takeda, Chugai, Kyowa Hakko Kirin, Taiho, and Mochida; personal fees and nonfinancial support from Yakult Honsha, Novartis, Bayer Yakuhin; and grants from Nippon Kayaku outside the submitted work. Dr Kang reports grants from ONO Pharmaceutical, AstraZeneca, and Eli Lilly. Dr Li reports grants from ONO Pharmaceutical and Bristol‐Myers Squibb. Dr Ferris reports grants and clinical trial support from AstraZeneca/MedImmune, Bristol‐Myers Squibb, Merck, Tesaro, and VentiRx Pharmaceuticals; advisory board/consultation fees from Amgen, AstraZeneca/MedImmune, Bain Capital Life Sciences, Bristol‐Myers Squibb, EMD Serono, GlaxoSmithKline, Iovance Biotherapeutics, Inc., Lilly, Merck, Numab Therapeutics AG, Oncorus, Inc., ONO Pharmaceutical Co. Ltd., Pfizer, PPD (Benitec, Immunicum), Regeneron Pharmaceuticals, Inc., Tesaro, Torque Therapeutics Inc., and TTMS. Dr Gillison reports grants from AstraZeneca, Bristol‐Myers Squibb, Kyowa, and Merck and personal fees from Amgen, AstraZeneca, Bristol‐Myers Squibb, Celgene, GlaxoSmithKline, Lilly, and Merck. Dr Tahara reports grants from ONO Pharmaceutical, Bristol‐Myers Squibb, Eisai, Otsuka, Boehringer Ingelheim, AstraZeneca, Pfizer, Novartis, and NanoCarrier and has received personal fees from ONO Pharmaceutical, Bristol‐Myers Squibb, Merck Sharp & Dohme, Bayer, Eisai, Otsuka, AstraZeneca, and Pfizer. T. Endo is an employee of ONO Pharmaceutical. V. Jayaprakash is an employee of Bristol‐Myers Squibb.

## Supporting information


**Figure S1** Swimmer plot in the Asian population
Abbreviations: N/A = not applicable; PR = partial response; SD = stable disease; PD = progressive disease

**Figure S2** Progression‐free survival (PFS) in the Asian population
Abbreviations: CI = confidence interval; HR = hazard ratio

**Figure S3** The change in tumor diameter over the time

**Figure S4** Post Progression Survival (PPS) in the Asian population
Abbreviations: CI = confidence interval; HR = hazard ratio

**Figure S5** Swimmer plot for the 11 Asian patients with skin reactions
Abbreviations: PR = partial response; PD = progressive disease

**Table S1** The details of 6 patients with partial response
