# Supplementary figures and images for: Two‐year follow‐up of a randomized phase III clinical trial of nivolumab vs. the investigator's choice of therapy in the Asian population for recurrent or metastatic squamous cell carcinoma of the head and neck (CheckMate 141)
Source: Head Neck. 2020 Jun 24;42(10):2852–62. doi: 10.1002/hed.26331 (PMC7540331; doi:10.1002/hed.26331)

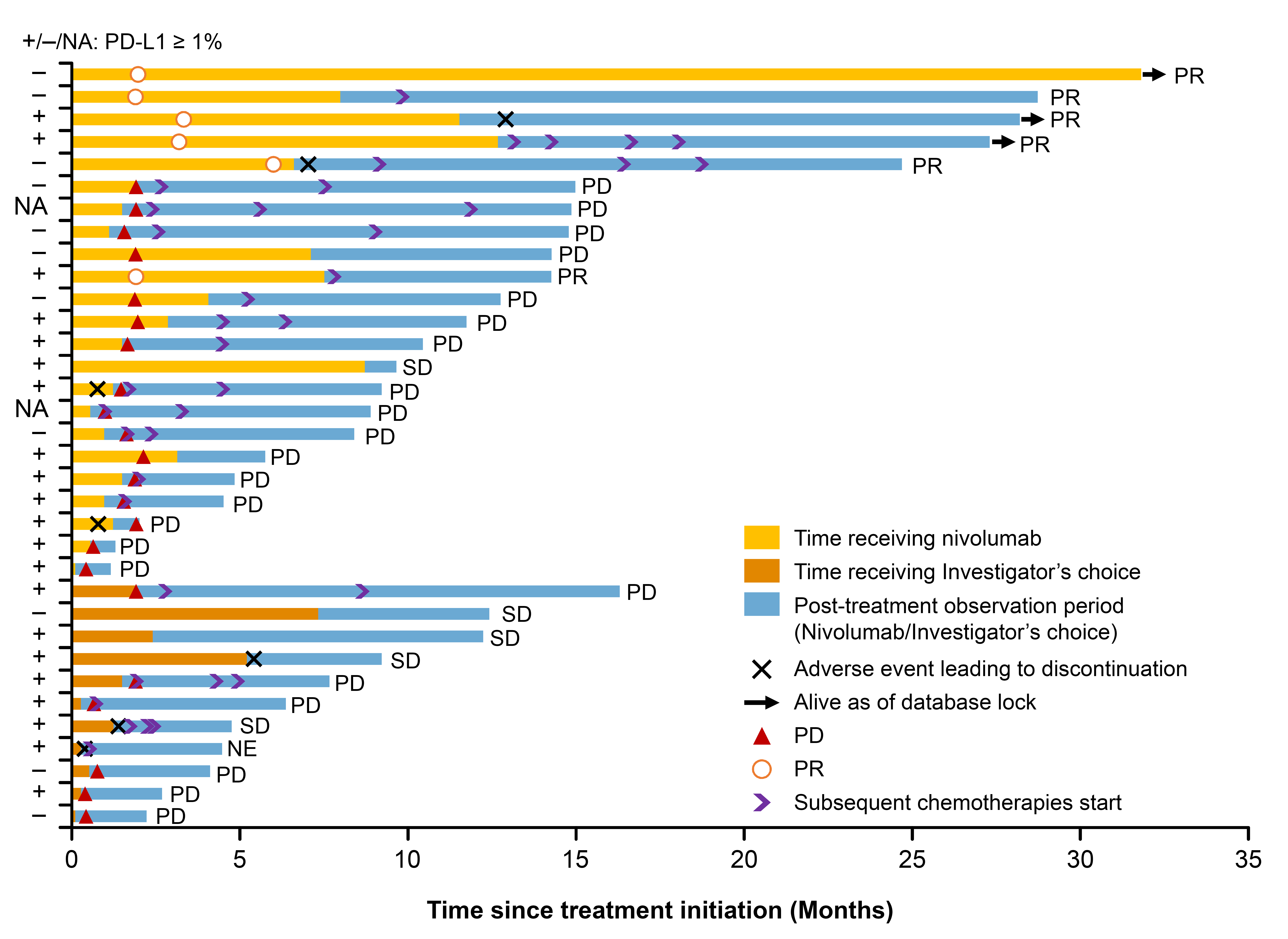

Supplement: Supplementary file 1 — Figure S1 Swimmer plot in the Asian population Abbreviations: N/A = not applicable; PR = partial response; SD = stable disease; PD = progressive disease [file HED-42-2852-s001.tif]

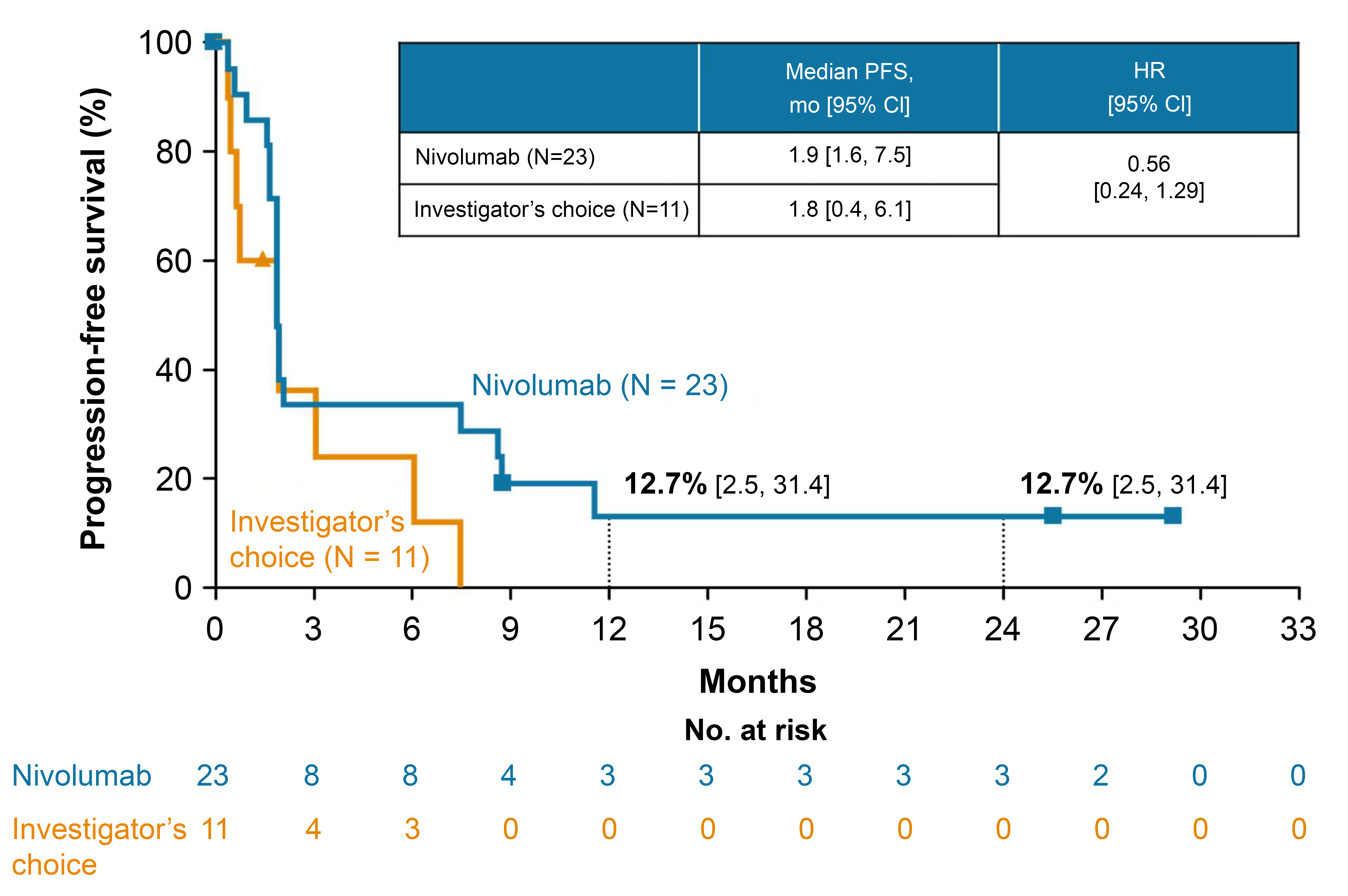

Supplement: Supplementary file 2 — Figure S2 Progression‐free survival (PFS) in the Asian population Abbreviations: CI = confidence interval; HR = hazard ratio [file HED-42-2852-s002.tif]

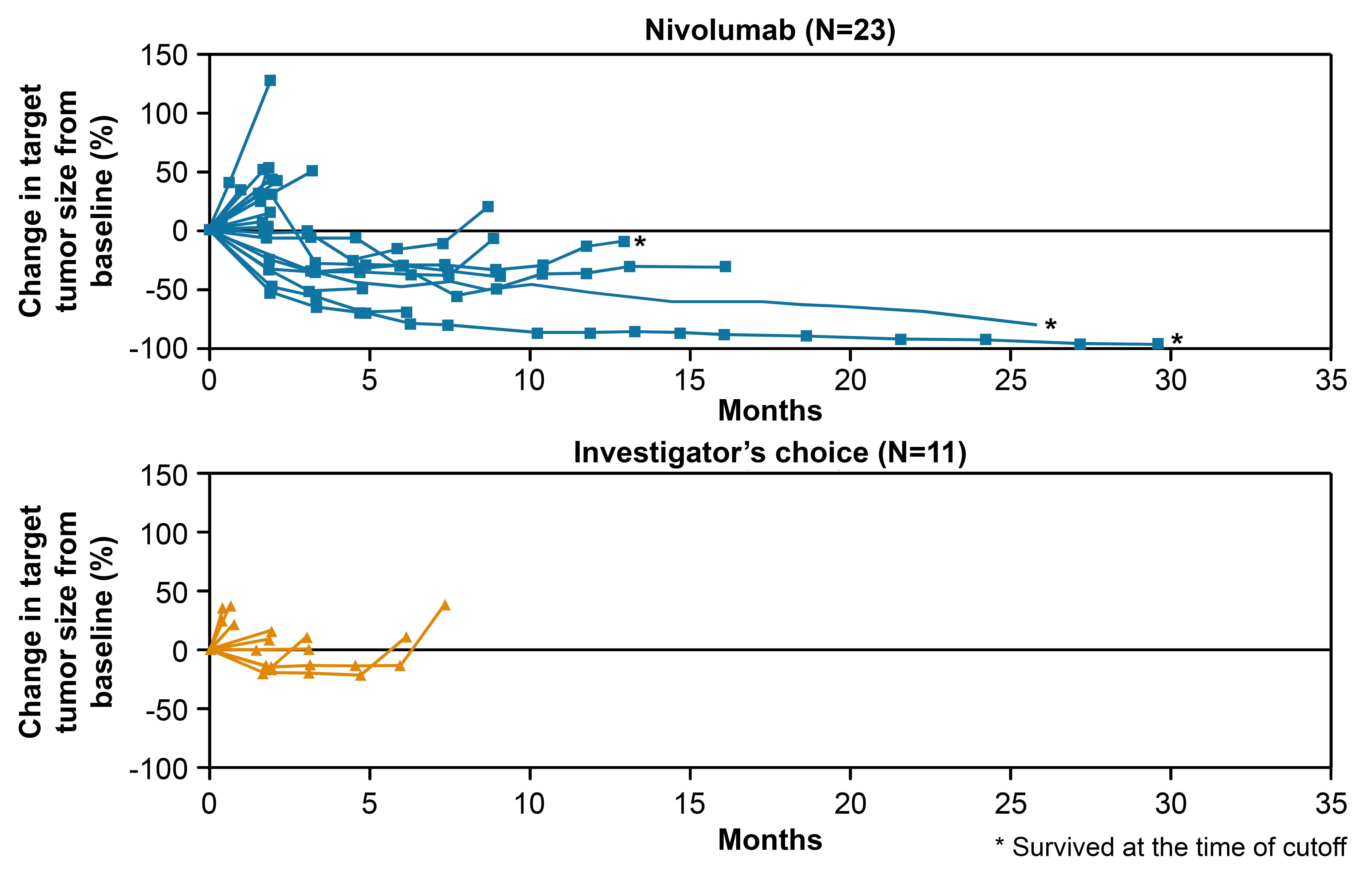

Supplement: Supplementary file 3 — Figure S3 The change in tumor diameter over the time [file HED-42-2852-s003.tif]

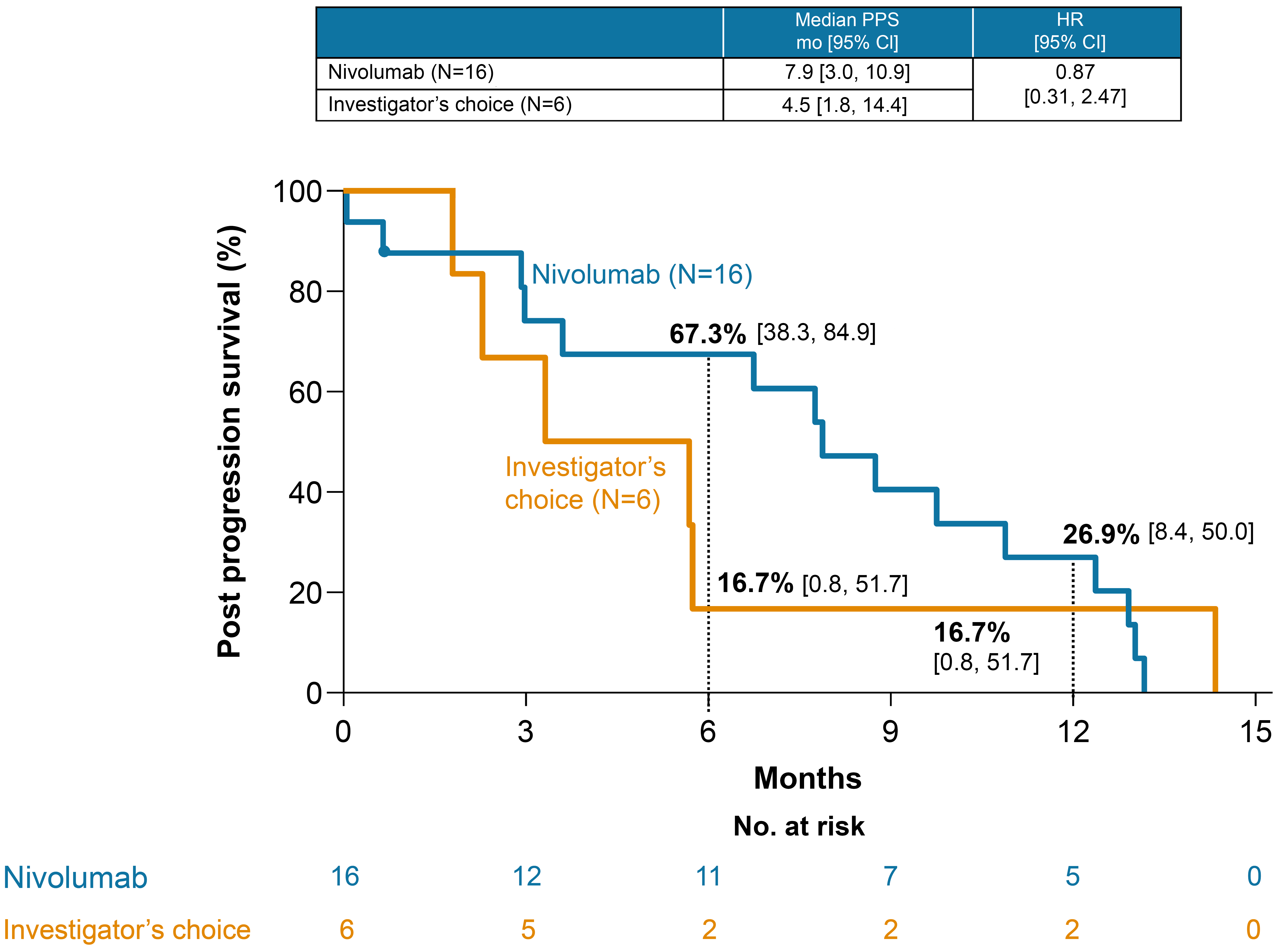

Supplement: Supplementary file 4 — Figure S4 Post Progression Survival (PPS) in the Asian population Abbreviations: CI = confidence interval; HR = hazard ratio [file HED-42-2852-s004.tif]

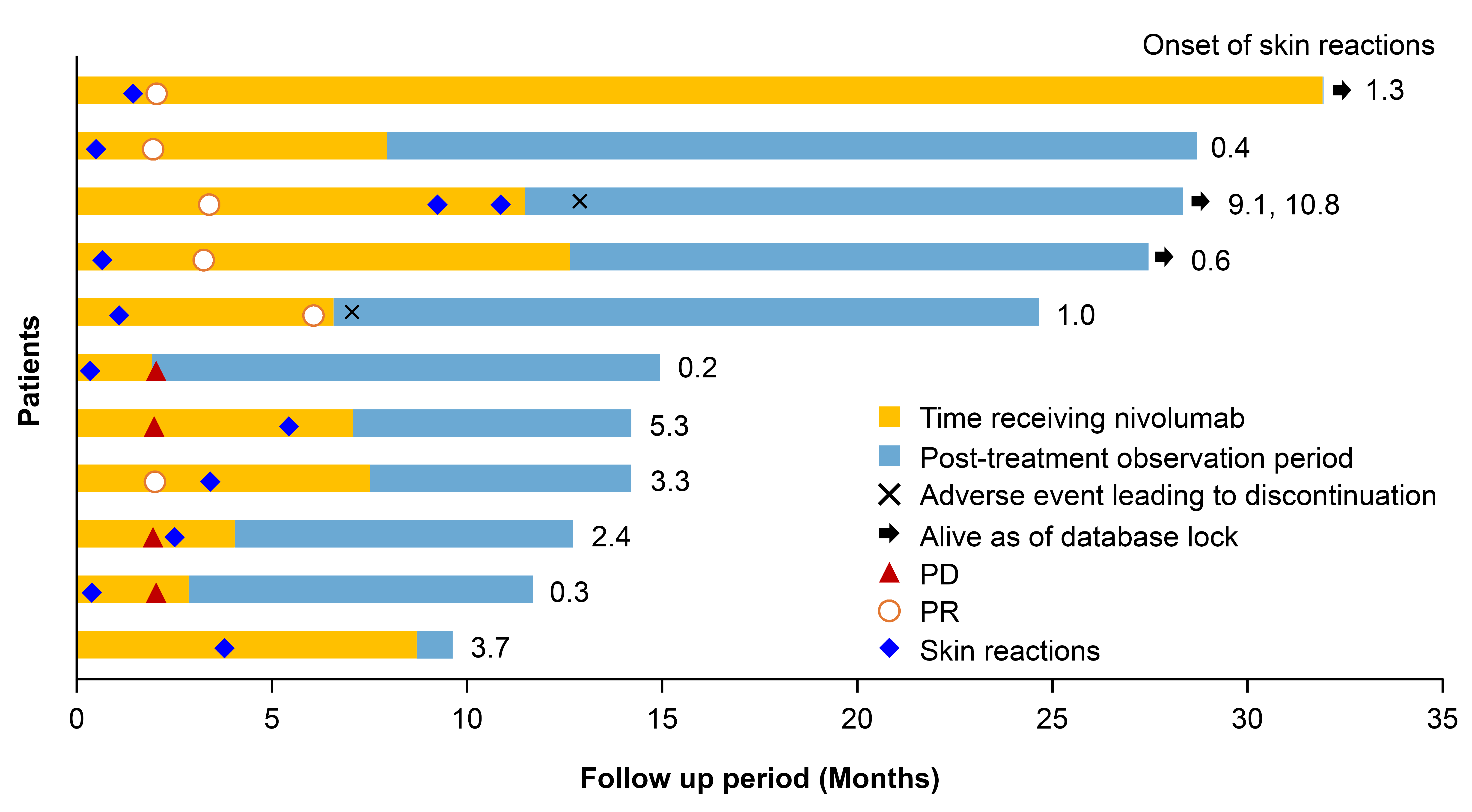

Supplement: Supplementary file 5 — Figure S5 Swimmer plot for the 11 Asian patients with skin reactions Abbreviations: PR = partial response; PD = progressive disease [file HED-42-2852-s005.tif]
