# Supplementary material for: Two‐year follow‐up of a randomized phase III clinical trial of nivolumab vs. the investigator's choice of therapy in the Asian population for recurrent or metastatic squamous cell carcinoma of the head and neck (CheckMate 141)
Source: Head Neck. 2020 Jun 24;42(10):2852–62. doi: 10.1002/hed.26331 (PMC7540331; doi:10.1002/hed.26331)
Supplement: Supplementary file 6 — Table S1 The details of 6 patients with partial response [file HED-42-2852-s006.docx]

**Supplementary Table 1**. The details of 6 patients with partial response

|  | Patient 1 | Patient 2 | Patient 3 | Patient 4 | Patient 5 | Patient 6 |
| --- | --- | --- | --- | --- | --- | --- |
| Sex | Male | Female | Male | Female | Male | Male |
| Age (years) | 61 | 53 | 65 | 59 | 42 | 61 |
| ECOG performance-status score | 1 | 1 | 1 | 0 | 1 | 1 |
| Site of primary tumor | PHARYNX | PHARYNX | ORAL CAVITY | ORAL CAVITY | PHARYNX | ORAL CAVITY |
| Previous lines of systemic cancer therapy | 1 | 5 | 2 | - | - | 1 |
| Tumor PD-L1 status^a^ | <1% | <1% | 70% | 2% | < 1% | 95% |
| Time since the initiation of nivolumab (months) | 32.0 | 28.7 | 28.4 | 27.5 | 24.7 | 14.2 |
| Time receiving nivolumab (months) | 31.9 | 7.9 | 11.5^b^ | 12.6 | 6.6^c^ | 7.5 |
| Follow-up time after nivolumab (months) | 0.03 | 20.8 | 16.9 | 14.8 | 18.1 | 6.7 |
| Regimen of chemotherapy after nivolumab | - | (I) PTX | - | (I) Pt  (II) Cmab  (III) UFT  (IV) Cmab | (I) UFT  (II) CDDP/5-FU  (III) Cmab | (I) TS-1 |

^a^Using the Dako PD-L1 IHC 28-8 pharmDx assay

^b^Treatment discontinuation due to deterioration of diabetes mellitus

^c^Treatment discontinuation due to pneumonia

Abbreviations: ECOG PS = Eastern Cooperative Oncology Group; PD-L1 = programmed death ligand 1; PTX = paclitaxel; Pt = cisplatin or carboplatin; Cmab = cetuximab; UFT = tegafur and uracil; CDDP = cisplatin; 5-FU = 5-fluoracil; TS-1 = tegafur, gimeracil, and oteracil
